# 

**Published:** 1904-04-15

**Authors:** 


### ANNOUNCEMENTS.

THE first annual meeting of the Montana State Dental Society was held in Helena, Montana, February 22d and 23d. The officers elected for the coming year are W. H. Barth, President; George Longway, Secretary. The second annual meeting will be held in Butte, Montana, February 20th, 21st, 1905.

---

THE fortieth annual meeting of the Illinois State Dental Society will be held at Peoria, Tuesday, Wednesday and Thursday, May 10th, 11th, and 12th. A splendid program including attractive and unusually interesting features is under course of preparation. The usual fare of one and one-third certificate plan will be obtained on all roads in the State and from St. Louis. Remember the date. All reputable practitioners cordially invited.

HART J. GOSLEE, *Secretary.*

---

THE Michigan State Dental Society will meet at Lansing, June 28th and 29th. An experiment is to be tried this year of presenting one subject at each session, by four different writers, each presenting a particular phase of the subject in a brief paper. The entire subject will then be opened for general discussion. As the meeting is to extend over two days, the six sessions will be fully occupied and no time will be wasted. A large number of clinics will be given and a good meeting is planned.

NORTHERN OHIO DENTAL ASSOCIATION will meet at Cleveland, June 7, 8 and 9, 1904,

---

BELIEVING that the greatest question before the profession and public today is how to render dental operations free from pain, the Executive Committee has arranged to make this session one of particular interest from the standpoint of those who desire to adopt such methods as will give their patients the greatest source of comfort while submitting to treatment at their hands. Each essayist has been requested to give that side of our work a prominent place in his paper and we have secured sufficient assurance from them to lead us to make the statement that pain-preventing methods will be thoroughly discussed. Clinicians have also been requested to demonstrate methods calculated to prevent suffering. To this end and in addition to the regular dental clinics there will be a symposium in the use of minor general anesthetics in surgery of the mouth, nose and throat, by some of the leading specialists in Cleveland. This does not mean that subjects of importance aside from pain-preventing methods will not be given the proper consideration, but simply that the former will be given the preference.

Positive that all men who desire to keep abreast of the times must keep in touch with the manufacturers in the progress made by them, every effort has been made to secure the largest and most complete exhibit ever shown in the State. By special arrangement, one entire floor of the Western Reserve Dental College will be devoted to exhibits, where ample space, water and electric power will be furnished and every opportunity will be afforded exhibitors to make their display one of the most attractive and interesting features of the meeting. Pledges received from some of the leading manufacturers and wholesale and retail houses up to date assure success in this undertaking. Several of them have signified their intention of sending special lecturers and demonstrators that the best possible understanding may be had of their goods.

Arrange your appointments and business affairs so that you can attend this meeting. Your attendance upon these meetings is a duty that you owe to yourself, your patient and your profession.

---

INTERNATIONAL DENTAL CONGRESS.—IT IS important that each member of the dental profession in America regard this effort to hold an International Dental Congress as a matter in which he has an individual interest, and one which he is under obligation to personally help toward a successful issue. The dental profession of America has not only its own professional record to maintain with a just pride, but, as it is called upon to act the part of host in a gathering of our colleagues from all parts of the world, it has to sustain the reputation of American hospitality as well.

The Committee of Organization appeals earnestly to each member of the profession to do his part in making the Congress a success. Later bulletins will be issued setting forth the personnel of the organization and other particulars, when the details have been more fully arranged.

The Congress will be divided into two departments: Department A—SCIENCE (divided into four sections). Department B—APPLIED SCIENCE (divided into six sections).

### DEPARTMENT A—SCIENCE.

1. Anatomy, Physiology, Histology and Microscopy: Chairman, M. H. Cryer, 1420 Chestnut St., Philadelphia, Pa.
2. Etiology, Pathology and Bacteriology: Chairman, R. H. Hofheinz, Chamber of Commerce, Rochester, N. Y.
3. Chemistry and Metallurgy: Chairman, J. D. Hodgen,

1005 Sutter St., San Francisco, Cal. 4. Oral Hygiene, Prophylaxis, Materia Medica and Therapeutics, and Electro-therapeutics: Chairman, A. H. Peck, 92 State St., Chicago, Ills.

DEPARTMENT B—APPLIED SCIENCE.

5. Oral Surgery: Chairman, G. V. I. Brown, 445 Milwaukee Ave., Milwaukee, Wis. 6. Orthodontia: Chairman, E. H. Angle, 1023 N. Grand Ave., St. Louis, Mo. 7. Operative Dentistry: Chairman, C. N. Johnson, Marshall, Field Building, Chicago, Ills. 8. Prosthesis: Chairman, C. R. Turner, 33d and Locust Sts., Philadelphia, Pa. 9. Education, Nomenclature, Literature and History: Chairman, Truman W. Brophy, Marshall Field Building, Chicago, Ills. 10. Legislation: Chairman, Wm. Carr, 35 West 46th St., New York, N. Y.

COMMITTEES.

Following are the committees appointed:

*Finance:* Chairman, C. S. Butler, 680 Main St., Buffalo, N. Y. *Program:* Chairman, A. H. Peck, 92 State St., Chicago, Ills. *Exhibits:* Chairman, D. M. Gallie, 100 State St., Chicago, Ills. *Transportation*: . (To be appointed.) *Reception:* Chairman, B. Holly Smith, 1007 Madison Ave., Baltimore, Md. *Registration:* Chairman, B. L. Thorpe, 3666 Olive St., St. Louis, Mo. *Printing and Publication:* Chairman, W. E. Boardman, 184 Boyleston St., Boston, Mass. *Conference with State and Local Dental Societies:* Chairman, J. A. Libbey, 524 Penn Ave., Pittsburg, Pa. *Dental Legislation*: Chairman, Wm. Carr, 35 West 46th St., New York, N. Y. *Auditing*: (Committee of Organization.) *Invitation:* Chairman, L. G. Noel, 527½ Church St., Nashville, Tenn. *Membership*: Chairman, J. D. Patterson, Keith and Perry Building, Kansas City, Mo. *Educational Methods*: Chairman, T. W. Brophy, Marshall Field Building, Chicago, Ills. *Oral Surgery:* Chairman, G. V. I. Brown, 445 Milwaukee

Ave., Milwaukee Wis. *Prosthetic Dentistry:* Chairman, C. R Turner, 33d and Locust Sts., Philadelphia, Pa. *Local Committee of Arrangements and Reception:* Chairman, Wm. Conrad, 3666 Olive St., St. Louis, Mo. *Essays:* Chairman, Wilbur F. Litch, 1500 Locust St., Philadelphia, Pa. *History of Dentistry:* Chairman, Wm. H. Trueman, 900 Spruce St., Philadelphia, Pa. *Nomenclature:* Chairman, A. H. Thompson, 720 Kansas Ave., Topeka, Kan. *Promotion of Appointment of Dental Surgeons in the Armies and Navies of the World:* Chairman, Wms. Donnally, 1018 14th St., N. W., Washington, D. C. *Care of the Teeth of the Poor:* Chairman, Thomas Fillebrown, 175 Newbury St., Boston, Mass. *Etiology, Pathology, and Bacteriology:* Chairman, N. H. Hofheinz, Chamber of Commerce, Rochester, R. Y. *Prize Essays:* Chairman, James Truman, 4505 Chester Ave., Philadelphia, Pa. *Oral Hygiene, Prophylaxis, Materia Medica and Therapeutics, and Electro-therapeutics:* Chairman, A. H. Peck, 92 State St., Chicago, Ills. *Operative Dentistry:* Chairman, C. N. Johnson, Marshall Field Building, Chicago, Ills. *Resolutions:* Chairman, J. Y. Crawford, Jackson Building, Nashville, Tenn. *Clinics:* Chairman, J. P. Gray, 24 Berry Block, Nashville, Tenn. *Nominations:* (To be appointed.) *Ad Interim* Chairman, G. V. I. Brown, 445 Milwaukee Ave., Milwaukee, Wis.

The rules provide that *membership in the Congress shall be open to all reputable legally-qualified practitioners of dentistry.* Membership in a State or local society is not a necessary qualification for membership in the Congress.

Each State Chairman is furnished with official application blanks and is authorized to accept the membership fee of ten dollars from all eligible applicants within his State. The State Chairman will at once forward the fee and official application with his indorsement to the chairman of the Finance Committee, who will issue the official certificate conferring membership in the Congress. No application from any of the States will be accepted by the Chairman of the Finance Committee unless approved by the State Chairman, whose indorsement is a certification of eligibility under the membership rules.

*A certificate of membership* in the Congress will entitle the holder thereof to all the rights and privileges of the Congress, the right of debate, and of voting on all questions which the Congress will be called upon to decide. It will also entitle the member to one copy of the official transactions when published and to participation in all the events for social entertainment which will be officially provided at the time of the Congress.

The attention of all reputable legally-qualified practitioners of dentistry is called to the foregoing plan authorized by the Committee of Organization for securing membership in the Congress, and the Committee earnestly appeals to each eligible practitioner in the United States who is interested in the success of this great international meeting to make application at once through his State Chairman for a membership certificate. By acting promptly in this matter the purpose of the committee to make the fourth International Dental Congress the largest and most successful meeting of dentists ever held will be realized, and the Congress will thus be placed upon a sound financial basis.

Let every one make it his individual business to help at least to the extent of enrolling himself as a member and the success of the undertaking will be quickly assured. Apply at once to your State Chairman. The State Chairman already appointed are:

*General Chairman:* J. A. Libbey, 524 Penn Ave., Pittsburg, Pa.

—✦—

THE Kentucky State Dental Association meets May 17th, 18th and 19th, 1904, at Louisville. Everybody invited.

—✦—

ILLINOIS State Board of Examiners will hold its meeting May 6th and 7th, at Chicago. Write Dr. J. G. Reid, 1204 Trude Building, Chicago, for particulars.

The Michigan State Examining Board will hold its semi-annual meeting in the Council Chambers at Grand Rapids, May 10th, 1904, at 9 a.m. Write Dr. W. C. McKinney, Saginaw, for particulars.

